# Brief Intervention in College Settings

**Published:** 2004

**Authors:** Mary E. Larimer, Jessica M. Cronce, Christine M. Lee, Jason R. Kilmer

**Affiliations:** Mary E. Larimer, Ph.D., is an associate professor in the Department of Psychiatry and Behavioral Sciences, University of Washington, Seattle. Jessica M. Cronce is a graduate student in the Department of Psychology, Yale University, New Haven, Connecticut. Christine M. Lee, Ph.D., is an acting assistant professor in the Department of Psychiatry and Behavioral Sciences, University of Washington, Seattle. Jason R. Kilmer, Ph.D., is an addictive behaviors specialist and adjunct member of the faculty at the Evergreen State College, Olympia, Washington, and a substance abuse prevention program coordinator at Saint Martin’s College in Lacey, Washington

**Keywords:** undergraduate student, alcohol abuse, binge drinking, heavy drinking, AODD (alcohol and other drug use disorder), identification and screening, interview, motivational interviewing, CAGE Questionnaire, Michigan Alcoholism Screening Test (MAST), Young Adult Alcohol Problems Screening Test (YAAPST), brief intervention, peer counseling, professional counseling, literature review

## Abstract

It is well established that college students have high rates of alcohol use and misuse and suffer the negative consequences of this behavior. Research evaluating the results of brief interventions with high-risk college students has shown these approaches to be successful in reducing alcohol consumption and/or related consequences. Several screening tools have been developed to detect the presence of problematic alcohol use and associated disorders, and some are designed specifically for use in a college student population. College campuses offer several opportunities to implement screening and interventions, including universal or large-scale assessments; health services, counseling centers, or local emergency rooms; or via established judicial or grievance systems set up to deal with students who violate campus alcohol policies. Issues to consider when implementing screening and brief interventions in college populations include who should deliver the interventions—peer or professional counselors—and how students should be encouraged to participate in the interventions. Regardless of how the measures are implemented, the content and process of the brief interventions should be based on the available scientific evidence regarding established efficacious interventions.

This article briefly summarizes the literature on college student drinking, the factors that can place students at risk for harmful consequences from their drinking, screening instruments and brief interventions shown to be effective with college students, and considerations and limitations in implementing such interventions. It concludes with clinical and research recommendations for further study of brief interventions in college populations.

## Epidemiology of Alcohol Use by College Students

The use and misuse of alcohol by young adult college students and the resulting negative consequences have been widely documented in the alcohol literature (see O’Malley and Johnston 2002 and Perkins 2002 for reviews). Several longitudinal and cross-sectional national studies have been tracking the use of alcohol among the nation’s youth and college students. In the 2003 Monitoring the Future (MTF) report, 86 percent of college students reported drinking alcohol at least once in their lifetime, and 66 percent reported drinking alcohol in the last month (Johnston et al. 2004).

The prevalence of college student drinking has been fairly stable over the past two decades, although tobacco and illegal drug use mainly have declined (Johnston et al. 2004). The pattern of alcohol use among college students is a serious cause for concern because many engage in heavy episodic, or binge, drinking, traditionally defined as having five or more drinks in a row^1^ (Johnston et al. 2004; Wechsler et al. 2002). Approximately 39 to 44 percent of college students reported binge drinking at least once in the 2 weeks prior to filling out the survey (Johnston et al. 2004; Wechsler et al. 2002). Additionally, according to one study, nearly one-third of college students met the criteria for alcohol abuse specified in the *Diagnostic and Statistical Manual of Mental Disorders, Fourth Edition* (DSM–IV), and 6 percent met its criteria for alcohol dependence (Knight et al. 2002).

Perkins (2002) provides a review of the negative physical, behavioral, legal, interpersonal, and institutional effects associated with alcohol use by college students. For example, many students who drink alcohol experience consequences such as physical illness (e.g., hangovers, nausea), academic impairment (e.g., missing class), and damage to others (e.g., getting into arguments/fights, vandalism). Students who engage in heavy episodic drinking also are at greater risk for experiencing these negative alcohol-related consequences (Wechsler et al. 2002).

### Natural History of Alcohol Use

Students who drink and drink heavily during high school have been found to continue similar drinking behavior while attending college. Longitudinal panel data from MTF found that during high school, college-bound seniors drink less than their non-college-bound peers (O’Malley and Johnston 2002). Although both groups increase their rates of heavy drinking after high school graduation, the rates for students in college increase more and actually surpass the rates of their noncollege peers. For members of both groups, the rate of heavy drinking peaks when they are around 21 or 22 and decreases steadily thereafter (Johnston et al. 2004). Many “mature out” of engaging in risk behavior, including alcohol use, when they attain adult roles and responsibilities (e.g., marriage, parenting, full-time employment) (Bachman et al. 1997).

**At a Glance**

**The Scope of Alcohol Problems on College Campuses**

- 86 percent of college students participating in the 2003 Monitoring the Future (MTF) study reported having consumed alcohol at least once in their lives (Johnston et al. 2004).
- 66 percent of college students in the 2003 MTF study reported drinking alcohol during the last month (Johnston et al. 2004).
- 39–44 percent of college students in the 2003 MTF study reported binge drinking at least once in the 2 weeks preceding the survey (Johnston et al. 2004).
- Almost one-third of college students studied met DSM–IV criteria for alcohol abuse; 6 percent met DSM–IV criteria for alcohol dependence (Knight et al. 2002).
- 32.5 percent of health centers at 4-year institutions routinely screen for alcohol problems (Knight et al. 2002).
- 17 percent of college health centers use standardized instruments in screening (Foote et al. 2004).

### Factors Associated With Alcohol Misuse in College

Research has shown that both individual and environmental factors are associated with increased risk for alcohol use and misuse. Among college students, individual factors such as a person’s family history of alcoholism, cognition (i.e., alcohol expectancies, drinking motives, perceived norms), and personality (i.e., impulsivity, extraversion, emotionality) are associated with alcohol use (see Baer 2002 for a review), as are involvement in fraternities or sororities and activities such as athletics (Baer 2002; Bartholow et al. 2003). Environmental factors that influence collegiate alcohol use include type of residence, college size and geographical region, and alcohol availability: Students who live on campus and in fraternities/sororities have higher rates of alcohol use and misuse; students attending larger colleges and colleges located in the Northeast and north central States tend to consume larger quantities of alcohol (Presley et al. 2002).

**Table:** Summary of the Research on Brief Intervention With College Students

Larimer and Cronce 2002	Assessment and brief feedback were associated with reductions in alcohol use and/or negative consequences. Three preliminary studies showed evidence of effectiveness of computerized (not in-person) feedback. Social marketing techniques may improve recruitment of students into alcohol prevention and intervention services.
Marlatt et al. 1998	High-risk students receiving a brief motivational feedback session (consisting of information on the student’s alcohol use, consequences, expectancies, and comparison of alcohol use with campus norms of use) reported reductions in use and consequences compared with control group participants. Changes were maintained at 2- and 4-year followups.
Borsari and Carey 2000	Heavy episodic drinkers (men: 5+ drinks, and women: 4+ drinks, at least twice a month) randomly assigned to motivational feedback interviews reported reductions in use at 6 weeks but no significant differences in number of negative consequences of use.
Larimer et al. 2001	Fraternity men randomly assigned either to a motivational feedback intervention or to a treatment-as-usual control group experienced similar reductions in use 1 year after the intervention.
Murphy et al. 2001	Heavier-drinking students receiving feedback interviews reported greater reductions in drinking than students assigned to education-only or assessment-only sessions.
McNally and Palfai 2003	Small-group normative feedback sessions were effective at reducing alcohol use among heavy-drinking students at 4-week followups.
Collins et al. 2002	Students receiving mailed feedback comparing their own drinking with their perceptions of drinking on campus reduced their alcohol consumption at 6 weeks, but not at 6 months, compared with control group students.
Murphy et al. 2004	Similar reductions in drinking were found for students who had received mailed motivational feedback and those who had had in-person feedback interviews.
Neighbors et al. 2004*a*	Students receiving computerized feedback about their drinking had reduced use at 3- and 6-month followups compared with an assessment-only control group.
O’Hare 1997	Mandating student violators of campus alcohol policy to screening and brief intervention may result in reaching a large number of at-risk students.
Borsari and Carey, in press	Students mandated to receive brief individual motivational sessions reduced their consumption more at 3 months and 6 months than did students mandated to receive standard alcohol education.
Barnett et al. 2004	Students who had a brief motivational feedback interview participated at higher rates in outside counseling than did students who had received an Internet-based educational intervention.
Larimer et al. 2001; O’Leary et al. 2002; Fromme and Corbin, in press	Individual skills-based or motivational enhancement interventions may be as effective in changing college students’ drinking behaviors when the interventions are provided by trained peer counselors as when they are provided by professionals, although the professionals may be more knowledgeable and have better skills.
Black and Coster 1996	Students who most need alcohol-related interventions may be least likely to participate in them.
Neighbors et al. 2004*b*	Drinking students on average were more interested in prevention programs than were non-drinkers. The more they typically drank, the higher their levels of interest and participation, but participation declined among the heaviest drinkers (8+ drinks per occasion).
Black and Smith 1994	Compared with the general population, heavier drinkers rated reminder contacts more important to getting them to attend intervention sessions.

## Advantages and Efficacy of Screening and Brief Interventions in College Populations

In response to the identified risk for and consequences of heavy episodic drinking by college students, the majority of college campuses have implemented some type of alcoholism prevention programming, and most of these efforts have focused on targeting individual drinkers through education or intervention. Larimer and Cronce (2002) reviewed individual intervention efforts from 1984 to 1999 and concluded that measures based on educational or awareness models had not been effective, although they found that the evidence of efficacy for brief motivational interventions was relatively strong. Specifically, seven studies with college or college-aged participants indicated that assessment and brief feedback, in individual or group intervention format, were associated with reductions in alcohol use, negative consequences, or both (see Larimer and Cronce 2002 for a review; for a summary of studies on brief interventions with college students, see the table).

For example, Marlatt and colleagues (1998) randomly assigned high-risk incoming freshmen to receive or not receive a 45-minute in-person motivational feedback session. The feedback sessions were based on assessment results that included feedback about their alcohol use, the consequences of their use, the students’ expectations regarding alcohol’s effects, and comparison of the students’ use with campus norms for alcohol consumption. Intervention group participants reported reductions in both use and consequences compared with control group participants, with changes maintained through 2-year and 4-year followups (Baer et al. 2001). Similarly, Borsari and Carey (2000) screened participants in an introductory psychology course and randomly assigned heavy episodic drinkers (men who had consumed five or more drinks or women who had consumed four or more drinks at least twice in the past month) to participate or not participate in a motivational feedback interview. Subjects in the motivational interviewing condition reported reductions in alcohol use at 6-week followup, although no significant differences were found in negative consequences between motivational interviewing and control conditions in this study.

Larimer and colleagues (2001) found similar effects in a sample of fraternity men randomly assigned to a motivational feedback intervention or a treatment-as-usual/educational control group. Larimer and colleagues included light and nondrinking fraternity members as well as heavier drinkers in the study. At the 1-year followup, the researchers found effects in this mixed sample similar to those found in heavy-drinking samples in other studies; this suggests that brief interventions can be used successfully with people who are not yet drinking heavily.

Recent studies have continued to support the efficacy of brief in-person motivational enhancement interventions. Murphy and colleagues (2001) found that heavier drinking students who received a feedback interview (i.e., a BASICS interview [see Dimeff et al. 1999]) reported greater reductions in drinking than did students assigned to an educational session or an assessment-only control group. In addition, one study supports the efficacy of small-group normative feedback methods for reducing alcohol use among heavy-drinking college students at 4-week followup (McNally and Palfai 2003).

Recent research increasingly has focused on using mailed or computerized feedback rather than in-person contact, and support for this strategy is growing. Larimer and Cronce (2002) found that three preliminary studies using this approach showed evidence of efficacy in reducing alcohol use during relatively short followup periods.

Studies published over the past 2 years have added to this literature and have shown generally positive short-term results. For example, Collins and colleagues (2002) compared students who received mailed, personalized, normative feedback (contrasting each student’s drinking with his/her perception of the typical student’s behavior and the actual norms for student drinking on campus) with those in a control group, and found reductions in drinking in the feedback group at 6 weeks compared with the control group participants, but not at 6 months. Murphy and colleagues (2004) compared the results of students who received mailed motivational feedback with those who had in-person feedback interviews and found similar reductions in drinking in both groups. Neighbors and colleagues (2004*a*) compared the results of a group that received computerized, personalized, normative feedback with results of an assessment-only control group and found reductions in alcohol use in the intervention group at 3- and 6-month followups.

Taken together, these studies provide strong support for the efficacy of brief, personalized, individual motivational feedback interviews, with mixed support for the implementation of normative or other feedback in group settings. In addition, support has been growing for feedback-based interventions delivered via the mail or computer/Internet rather than in person, at least in promoting short-term drinking reductions. Questions remain regarding long-term efficacy of this strategy.

## Special Considerations and Challenges to Screening and Brief Intervention in College Populations

Studies evaluating the impact of brief interventions with high-risk college drinkers have demonstrated the success of these interventions in reducing alcohol consumption and/or related consequences. The keys to effective dissemination of this approach are to identify the students who should receive interventions and then deliver a brief intervention as needed.^2^ This section examines the appropriate standardized measures to use in screening programs on college campuses, possible settings for implementing screening programs, and considerations involved in identifying practical, ethical, and cost-effective methods for providing intervention.

### Screening Tools

Choosing appropriate measures is vital for implementing successful alcohol screening on college campuses. Factors to consider in making this choice include the severity and temporal stability (i.e., transient versus chronic) of the problem being assessed, the ability of the measure to detect accurately the problem within the population of interest, and the feasibility of implementation (which depends on the measure’s length, format, and cost, given time, personnel, and budgetary constraints). Although several screening tools have been developed to detect the presence of problematic alcohol use and associated disorders, this article addresses only those that are widely used or specifically designed for a college student population (see the textbox “Screening Instruments Used With College Students”). All the measures discussed here can be completed via interview or self-administration, and most are available free of charge.^3^

**Screening Instruments Used With College Students**

**Table:** 

**CAGE**	Criticized for lacking sensitivity to detect the range of drinking problems experienced by college students
**MAST**	When used with college students, a cutoff of 7 on the full version resulted in perfect sensitivity and 87.7-percent specificity, but because MAST focuses on more advanced or stable alcohol problems, its usefulness with college students may be limited
**CAPS-r**	Good reliability and validity when used with a general college student population
**AUDIT**	The cutoff score appropriate for college students is a matter of disagreement. A study using DSM–III criteria for alcohol abuse and dependence showed adequate sensitivity for a cutoff score of 11. A study using DSM–IV criteria found a similar degree of sensitivity using a cutoff score of 6. Whichever criteria were used, the test overidentified problem drinkers at a high rate.

Screening tools that identify problems experienced over the course of the person’s lifetime include the CAGE questionnaire, the Michigan Alcoholism Screening Test (MAST), and the Young Adult Alcohol Problems Screening Test (YAAPST). The CAGE is a four-item measure that takes approximately 1 minute to complete.^4^ The CAGE has been used with college student populations but has been criticized repeatedly for lacking adequate sensitivity to detect the constellation of drinking problems experienced by people in this age group (see Larimer and Cronce 2002 for further discussion and relevant references).

Versions of the MAST are available that include from 9 to 25 items; the longest version takes less than 10 minutes to complete. One study using a convenience sample^5^ of college students found that a cutoff score of 7 on the full version of the MAST resulted in perfect sensitivity (100 percent) and reasonable specificity (87.7 percent) when compared with a score of 14 or greater on the Alcohol Dependence Scale (ADS), the criterion used to denote the presence of alcohol dependence (Martin et al. 1990). However, the MAST’s focus on detecting more advanced and/or stable problems with alcohol (such as dependence) may limit its predictive utility within this population (Svanum and McGrew 1995). In addition, the wording of items that relate to family history of alcohol problems may cause light drinkers or nondrinkers to over-identify themselves as problem drinkers (Svikis et al. 1991).

The YAAPST consists of 27 items, which, along with a full description of its psychometric properties, are published in the source document (Hurlbut and Sher 1992). The YAAPST takes less than 10 minutes to complete and has demonstrated good reliability (internal consistency and test–retest) and validity (construct, concurrent, and criterion), with reasonable sensitivity and specificity (92 percent and 57 percent, respectively, using a cutoff score of 4). The YAAPST, along with four other screening tools—the College Alcohol Problems Scale–revised (CAPS-r), the Rutgers Alcohol Problem Index (RAPI), NIAAA’s alcohol consumption question sets, and the Alcohol Use Disorders Identification Test (AUDIT)—can be used to detect alcohol problems experienced in the past year.

The CAPS-r is an eight-item assessment that takes approximately 3 minutes to complete. It has demonstrated good reliability^6^ and concurrent validity in a general college student population; total score on this test correlated significantly with various indexes of alcohol consumption (correlation coefficients [*r*] ranged from .44 to .51) and with the YAAPST (*r* = .78)^7^ (Maddock et al. 2001).

Two versions of the RAPI currently are in use—the original 23-item version and a briefer 18-item version—both of which can be completed in less than 10 minutes. The RAPI has shown good internal consistency (Neal and Carey 2004) and test–retest reliability using 1-, 6-, and 12-month time-frames (Miller et al. 2002), and it has been correlated significantly with a composite of drinking frequency, typical quantity, and frequency of intoxication (*r* ranged from .35 to .57) (White and Labouvie 1989).

Questions about consumption also can be used as a screening tool for alcohol problems. NIAAA produced four sets of questions, which are currently used for research and not implemented in clinical settings. Ranging from three to six items, from shortest to longest, they provide an increasingly detailed picture of a person’s drinking pattern. The three-item version takes less than 5 minutes to complete and includes questions on the frequency of drinking occasions over the past 12 months, the typical amount consumed, and the number of binge episodes (see footnote 1 regarding NIAAA’s definition of a binge episode) (NIAAA 2003). Although these questions have not yet been used specifically for screening, people who endorse one or more of the behaviors assessed by these questions (i.e., binge drinking and frequent alcohol use) have been shown to be at high risk for experiencing alcohol-related problems and therefore would be appropriate candidates to receive prevention or intervention.

The AUDIT is a 2-minute-long, 10-item assessment. The appropriate cutoff score to use for screening college students has been disputed. One study using DSM–III criteria for alcohol abuse and dependence has shown adequate sensitivity at a score of 11 (84 percent) (Fleming et al. 1991), whereas another study using DSM–IV criteria found that a cutoff score of 6 led to a similar degree of sensitivity (80.2 percent) (Aertgeerts et al. 2000). Both cutoff scores resulted in a high degree of overidentification (29 percent and 22 percent false positives, respectively).

A more recent study (Kokotailo et al. 2004) using high-risk drinking as the criterion^8^ suggested that a cutoff score of 8 results in levels of sensitivity (82 percent) and specificity (78 percent) comparable to the earlier studies. Use of this cutoff score, however, may be more appropriate for universal screening efforts within this population, given the greater prevalence of high-risk, heavy episodic alcohol consumption relative to clinically diagnosable alcohol use disorders.

When trying to identify people at risk within the student body as a whole, a screening tool is used most appropriately as the first step in a multistage assessment process. Thus, selecting a screening tool and corresponding threshold that errs on the side of identifying a larger percentage of people is desirable. Those who initially screen positive can be given additional assessments to determine the nature and extent of their problem, and this information can be used to generate feedback for brief intervention as well as to make appropriate referrals for additional services if indicated.

### Screening Implementation

College campuses offer several opportunities to implement screening and intervention. Although screening for risky alcohol use could and often does occur in health and counseling settings, this allows for the screening of only those students seeking these services on campus.

#### Universal Screening

With hopes of identifying risky drinking practices early, large questionnaire-based screening efforts that include measures of quantity, frequency, and consequences could be used to identify and refer students for brief interventions. Clearly the most ambitious screening approach, depending on campus size, this could involve annually screening all students or, as a high-risk group, all incoming first-year students.

Colleges and universities may be familiar with campuswide assessments using anonymous surveys (e.g., for compiling data for use with social norms campaigns), but a controlled study of implementation efforts with identifiable participant data for referral purposes has not been done outside of an efficacy research context. Theoretically, an assessment outside of the research context could be implemented with first-year students during required orientation activities or, with the incoming class, in identified residence groups (e.g., fraternities, sororities, residence halls). Casting the widest net for screening purposes has the advantage of serving as the basis for a large-scale universal or targeted prevention program using feedback from the assessment.

A potential disadvantage to a campus-wide assessment or screening of all first-year students is that this approach may create distrust in students regarding the intent or purpose of the screening. As college student drinking continues to receive attention in the popular press, students may be suspicious of an assessment or screening if they fear it signals a “crackdown” or is intended to identify the “troublemakers.” Further, if students learn that a “high score” results in a referral for alcohol-related counseling or other interventions, they may attempt to avoid taking part. Collectively, these concerns could lead students to report their behavior inaccurately (particularly in cases of underage drinking). Sampling strategies and expectations would need to be carefully defined, given that, for research purposes, a representative sample (in lieu of an entire student population) usually is targeted, participation is entirely voluntary, and a participant can choose to leave any number of questions unanswered.

Another potential concern is institutional liability. That is, questions of liability or responsibility could theoretically arise if the campus collects information on students’ illegal or risky behavior but does not intervene, particularly in the event that a medical emergency or judicial situation later arises. Confidentiality (e.g., in regards to the Family Educational Rights and Privacy Act governing confidentiality and privacy issues related to college students’ academic records) also is a concern, especially regarding who should have access to the screening information and for what purposes, if any, it will be used other than for intervention or referral.

Finally, the cost of screening all students could be prohibitive, depending on campus size. Zarkin and colleagues (2003) note that a possible barrier to implementing prevention efforts may be the one-time cost associated with startup as well as the costs related to technical assistance. All these issues are surmountable, but they require careful consideration and consultation to resolve.

#### Screening in Health Care and Counseling Facilities or Emergency Rooms

Screening may occur in the campus health center, counseling center, or local hospital emergency room (e.g., students can answer questions as part of normal intake procedures). The results of screening in these settings could set the stage for a brief intervention or referral for services. Several advantages to this approach exist. Research has demonstrated that empirically supported brief interventions can be successfully extended into campus emergency rooms and health centers (Dimeff and McNeely 2000; Helmkamp et al. 2003). Further, trained medical, nursing, and mental health staff are available to provide or supervise the screening and intervention process in the course of their ordinary interactions with patients, and issues relating to confidentiality of the screening information are clearer in these settings. Screening in these facilities is effective not only for identifying students with at-risk or problematic alcohol use but also for identifying symptoms related to a student’s presenting problems or health concerns. This information then ultimately can be used to aid in treatment planning.

Limitations to conducting screening and intervention in student health settings include possible resistance on the part of health care providers or staff to implementing screening and brief alcohol interventions in this context (given the time and effort required, and staff ’s possible lack of comfort with providing alcohol-related interventions, as well as the fact that the majority of students will present for problems other than drinking and providers may be uneasy about switching the focus). Similarly, students may not be willing to discuss or be motivated to change their drinking behavior in the face of other, more salient, presenting problems.

In addition, screening and intervention in student health facilities will miss those students who seek their medical or mental health services outside the campus health care system, as well as students who never seek such services and experience no health-related consequences of their drinking despite being at increased risk for other consequences. It is important, too, that the screening assessment used by health care providers measure the behavior of interest adequately and appropriately. Foote and colleagues (2004) found, for example, that although 32.5 percent of health centers at 4-year institutions routinely screened for alcohol problems, only 17 percent of these reported use of standardized instruments as part of their screening.

#### Screening as Part of Campus Judicial or Grievance Systems

Incorporating screening into campus judicial systems has several advantages. First, many campuses already have policies in place that require students cited for alcohol policy violations to complete assessment and interventions (Anderson and Gadaleto 2001), and trained staff typically are available to respond to these policy violators. As a result, incorporating systematic screening and brief interventions into this model may encounter fewer barriers caused by lack of campus resources than other wide-scale approaches. In addition, research suggests that students mandated to attend a program because of an alcohol policy violation are, on average, at higher risk for experiencing other alcohol-related negative consequences (O’Hare 1997), suggesting screening and brief intervention in this setting may reach a large number of at-risk students.

Further, the use of systematic screening in this setting may help to triage policy violators. For example, students who violated policy but did not drink to excess (e.g., students who were mandated to attend the program because they were at a party at which other people were drinking heavily and caught the attention of campus police) might receive less time- and resource-intensive interventions than those whose drinking already is excessive or who are showing indications of alcohol dependence and need for treatment.

Unfortunately, research on the effectiveness of screening and brief interventions with mandated students is limited. Larimer and Cronce (2002) identified only a single controlled study focusing on mandated students during the 15-year period from 1984 to 1999 and found no studies of brief motivational interventions in this population. Fortunately, more recently, several researchers have devoted increased efforts to evaluating other types of interventions for mandated students, and some encouraging findings are emerging. Fromme and Corbin (in press) reported comparable efficacy for mandated and voluntary students who participated in a two-session group lifestyle management class (LMC) that combined normative, skills-based, and motivational elements. Compared with students in the assessment-only control group, LMC participants reported significantly reduced incidents of driving after drinking, and male LMC participants in the mandated group showed greater reductions in heavy-drinking behavior.

Borsari and Carey (in press) compared a brief individual motivational intervention with standard alcohol education for mandated students and found the brief motivational intervention produced greater reductions in alcohol-related problems at 3- and 6-month followups. Barnett and colleagues (2004) reported preliminary findings on these interventions as well as their own preliminary data suggesting that students who had a brief individual motivational feedback interview participated at a higher rate in further outside counseling compared with students who had a computer-based educational intervention. These findings suggest screening and brief intervention can be efficacious for mandated students, but additional research is needed to more fully explore the conditions under which these interventions are most helpful.

In addition to concerns regarding efficacy, a potential limitation of screening and brief intervention with mandated students (as with health care settings) is the focus on a small and potentially nonrepresentative sample of the population. Further, compared with students involved in campuswide screening, those screened in a judicial setting may be even more suspicious of the reasons for screening and mistrustful of how the information will be used. To reduce suspicion and defensiveness, providers might assure students that the results of the screening will not impact whatever sanctions are imposed on them and will not be provided to campus administrators or in any way be used in a punitive manner.

### Intervention Providers and Student Participation in Receiving Interventions

Two issues to consider when implementing screening and brief interventions in college populations are who should deliver the interventions—peer or professional counselors—and how students should be encouraged to participate in the interventions.

#### Peer vs. Professional Providers

Training student peers to provide the brief interventions is one way to provide efficacious brief interventions on campus while reducing costs. This method has potential advantages and disadvantages. An advantage is that peer counseling often is used on college campuses for academic and health problems and generally has been regarded as effective in these circumstances (see Cuijpers 2002 for a review). Although few researchers have empirically evaluated the effectiveness of peer-provided individual skills-based or motivational enhancement interventions, recent research indicates that, on average, trained peer counselors (i.e., college undergraduates) are as effective as professionals in encouraging drinking changes among college students (Larimer et al. 2001; O’Leary et al. 2002; Fromme and Corbin, in press). These findings suggest that using peer counselors may improve the feasibility of universal or targeted brief interventions with college students once screening and assessment have indicated their drinking behavior warrants such attention.

A disadvantage is that participants and outside raters typically find professional providers are more knowledgeable and have better skills than peer counselors (Fromme and Corbin, in press), so some aspects of the intervention may suffer when delivered by peers. In addition, peer providers require training and supervision; most research protocols provide weekly individual or group supervision by a trained therapist.

#### Encouraging Students to Participate in Screening and Intervention

A challenge to overcome, especially for interventions delivered outside the health center and mandated contexts, is how best to encourage students to participate in screening and brief interventions. Some research suggests that those students who most need alcohol-related interventions may be least likely to participate (Black and Coster 1996). More recent research suggests that, on average, drinking students are more interested in prevention programs than are nondrinking students (Neighbors et al. 2004*b*), and their interest and participation in alcohol-consumption prevention activities increased as their typical rates of drinking increased. However, this research also indicates that participation rates declined for the very heaviest drinkers (those consuming seven to eight or more drinks per typical occasion) in comparison with students who drank at lower levels. These findings suggest the need to improve methods to increase participation in brief interventions.

One method for encouraging participation may be to treat students as consumers of brief intervention services and to market the intervention product and attend to customer concerns in development and implementation. Larimer and Cronce (2002) reviewed research suggesting that social marketing techniques may improve recruitment of students to alcoholism prevention and intervention services.

Calling students when they miss appointments and using other program reminders also may increase participation by heavier drinkers. Black and Smith (1994) found in one study that heavier drinkers rated reminder contacts as more important in getting them to attend intervention sessions than did people in the general population.

## Conclusions and Recommendations

Several conclusions and recommendations arise from this review. First, multiple independent studies using different intervention protocols and implementation strategies indicate that screening and brief intervention are effective for reducing college student drinking, although the duration, magnitude, and exact nature of the effects vary. Thus, unlike many interventions for college drinking, college administrators can trust that a carefully implemented program of screening and brief intervention will work (Larimer and Cronce 2002). Research provides the strongest support for interventions that are delivered to students individually and include personalized feedback about norms, expectations, and risks associated with alcohol consumption. There also is emerging support for the efficacy of mailed or computerized feedback alone in producing at least short-term reductions in students’ alcohol consumption.

College administrators contemplating the use of universal screening programs should consider the following issues:

- Who will have access to the data
- What liability and confidentiality issues may arise
- Who will intervene once the screening data are collected.

One recommendation is to provide institutional support for a provider in either health or counseling services who would be identified as a specialist in this area, thus reducing the threshold for access to these services and the need for referral to off-campus providers. This person could coordinate screening in all settings—in the health or counseling center, in judicial settings, and campuswide—which would solve some of the confidentiality issues raised by the involvement of academic affairs offices in screening. Using volunteer or hourly paid peer providers who are trained and supervised by this qualified professional offers a cost-effective means for providing brief interventions to more students.

Campus administrators also might consider a stepped-care approach to implementing brief interventions (Breslin et al. 1999). Such an approach could begin with assessment and provision of feedback via the Internet (Kypri et al. 2003), moving to in-person intervention only for those students who are identified as having more severe alcohol-related problems or those who do not respond to lower-intensity interventions. In addition, it may be advisable to include assessments for determining the use of other drugs of abuse and other psychiatric and health symptoms to place the brief alcohol intervention in context with other health risk behaviors.

Finally, the content and process of the brief interventions offered on college campuses should be based on the available scientific evidence regarding established effective interventions, as reviewed in this article and elsewhere (Larimer and Cronce 2002). To the extent that barriers to implementation require adapting these strategies to meet campus needs, administrators should evaluate the impact of these modifications and collect both satisfaction and behavioral outcome data. Further evaluation of the relative efficacy of in-person, mailed, or computerized brief interventions is necessary as well as comparison of these interventions with other individual and environmental interventions for college drinking. Ultimately, a combination of strategies may be necessary to address college student drinking, with screening and brief intervention constituting one component.

## Footnotes

1 The National Institute on Alcohol Abuse and Alcoholism (NIAAA) defines binge drinking as the amount of alcohol leading to a blood alcohol content (BAC) of 0.08, which, for most adults, would be reached by consuming five drinks for men or four for women over a 2-hour period (NIAAA 2004).

2 Screening can be differentiated from assessment in that the purpose of the former is to identify people at risk for or actually experiencing harm associated with their drinking, and the purpose of the latter is to gather detailed information about people’s patterns of consumption and consequences to inform intervention (Connors and Volk 2003). Some of the measures mentioned in this section can be used for both screening and assessment; however, the authors’ discussion of them here is limited to their utility as screening tools.

3 Copies of the CAGE and AUDIT are included in the “Screening Tests” sidebar on pages 78–79 of this issue. These instruments as well as the CAPS-r and RAPI also can be found in the second edition of *Assessing Alcohol Problems: A Guide for Clinicians and Researchers* (Allen and Wilson 2003) along with references to source documentation and other articles relevant to each assessment’s psychometrics. A copy of the MAST can be purchased for $40 from the author of the assessment (see Allen and Wilson 2003 for contact information) but may be used without further fee. The YAAPST and NIAAA’s alcohol consumption question sets also can be used at no cost; each measure is published in its entirety in the source documentation cited in the text.

4 Time estimates for completion of each measure are based, in part, on information from Allen and Wilson (2003).

5 A convenience sample consists of participants chosen at will by the investigators, rather than by another preset selection scheme, such as choosing consecutive patients. Selecting participants in this way may introduce some bias into the sample.

6 Cronbach’s coefficient alpha = 0.79 for the personal problems subscale and 0.75 for the social problems subscale.

7 The correlation coefficient (*r*) indicates the magnitude and direction of the linear relationship between two variables. Values of *r* that are closer to zero indicate a weaker association, and values that are closer to 1 (either positive or negative) indicate a stronger association. Positive values of *r* represent a linear increase in one variable as the other also increases, whereas negative values indicate a linear increase in one variable as the other decreases.

8 In the Kokotailo et al. study, high-risk drinking was defined as: for men, consuming 5 or more consecutive drinks on 4 or more occasions, or 57 or more drinks total during the preceding 28-day period; or for women, consuming 4 or more consecutive drinks on 4 or more occasions, or 29 or more drinks total during the preceding 28-day period.
